# Applications of Melatonin in Female Reproduction in the Context of Oxidative Stress

**DOI:** 10.1155/2021/6668365

**Published:** 2021-07-29

**Authors:** Yonghui Jiang, Huangcong Shi, Yue Liu, Shigang Zhao, Han Zhao

**Affiliations:** ^1^Center for Reproductive Medicine, Cheeloo College of Medicine, Shandong University, Jinan 250012, China; ^2^Key Laboratory of Reproductive Endocrinology of Ministry of Education, Shandong University, Jinan 250012, China

## Abstract

Oxidative stress has been recognized as one of the causal mediators of female infertility by affecting the oocyte quality and early embryo development. Improving oxidative stress is essential for reproductive health. Melatonin, a self-secreted antioxidant, has a wide range of effects by improving mitochondrial function and reducing the damage of reactive oxygen species (ROS). This minireview illustrates the applications of melatonin in reproduction from four aspects: physiological ovarian aging, vitrification freezing, *in vitro* maturation (IVM), and oxidative stress homeostasis imbalance associated with polycystic ovary syndrome (PCOS), emphasising the role of melatonin in improving the quality of oocytes in assisted reproduction and other adverse conditions.

## 1. Introduction

Oocyte and early embryo development have a compelling link with oxidative stress, which have been reviewed for their devastating consequences on oocyte quality and female fertility [1–5]. How to reduce oxidative stress has become an important strategy in reproductive health.

Melatonin (N-acetyl-5-methoxytryptamine), an indoleamine synthesized and secreted by the pineal gland, is distributed in various tissues, including the female reproductive system [6, 7]. And it is well known for its function in the circadian sleep-wake cycle [8]. As a powerful antioxidant and free radical scavenger, melatonin also has multiple benefits for reproduction, such as antioxidative stress in germ cells, delay of ovarian aging, promotion of oocyte maturation, and augment of embryo development [9–12]. It eliminates free radicals and has significant antioxidant effects in two ways [7]. One acts as an antioxidant and regulates ovarian function by binding to receptors (melatonin receptor 1, MT1, and melatonin receptor 2, MT2, [13]), which is associated with decreased downstream molecules, such as cyclic adenosine monophosphate (cAMP) and cyclic guanosine monophosphate (cGMP), as well as increased phospholipase C (PLC) [14]. In addition, melatonin can directly chelate oxygen and nitrogen reactive species as well as mobilize the intracellular antioxidant enzyme without its receptors [15].

Based on the physiological role of melatonin, we reviewed the applications of melatonin in reproduction from four aspects: delaying physiological aging of ovaries, ameliorating oocyte quality during vitrification freezing, promoting oocyte development during assisted reproductive technology (ART), such as *in vitro* maturation (IVM), and reducing pathological oxidative stress in polycystic ovary syndrome (PCOS).

## 2. Melatonin Delays Ovarian Aging

Female fertility progressively declines with age irreversibly, peaking at 25, declining by 35, and falling rapidly after 40 [16, 17]. This deterioration in fertility is closely linked to progressive ovarian aging, characterised by decreased oocyte quality as well as quantity, and the relationship between oocyte aging and oxidative stress is now well elaborated in detail. Oxidative stress, caused by ROS, is an important factor of physiological ovarian aging and telomere activity [18]. And ROS level in the follicular fluid can be used as biochemical markers to measure ovarian aging and follicular metabolic age [19]. Further analysis based on single-cell transcriptomics and cell-type-specific aging-associated transcriptional changes revealed the interference of specific antioxidant signals to early stage oocytes and granulosa cells, which suggested that oxidative damage is a pivotal factor in the decline of ovarian function with age [20]. Indeed, increased total antioxidant capacity (TAC) and superoxide dismutase (SOD) as well as decreased malondialdehyde (MDA) contributes to improve the quality of oocytes in aged mice [21].

Additionally, high expression of the autophagy-associated proteins (light chain protein 3, LC3, etc.) is also related to oxidative stress damage [22, 23]. Researchers have demonstrated that the mRNA levels of *LC3a* and *LC3b* significantly reduced in melatonin-treated aged mice (43 weeks), indicating that melatonin could delay fertility decline through autophagy [23]. Mitochondrial function degrades with age, adversely affecting ovarian reserves, and triggers ovarian aging [24]. Relative studies have shown that melatonin can reduce ROS level and maintain mitochondrial membrane potential (MMP) in cultured aging oocytes via MT1/AMPK pathway *in vitro* [21]. Furthermore, melatonin treatment can reverse ovarian aging caused by gene inner mitochondrial membrane peptidase 2-like (*Immp2l*) mutant through blocking the ROS-Wnt/*β*-catenin estrogen pathway [25].

The remarkable anti-aging effects of melatonin have also been closely linked to the sirtuins, a classical family of proteins with NAD^+^-dependent deacetylase or mono-ADP-ribosyltransferase activity, which play an important role in protecting oocytes from oxidative damage and aging [26]. Melatonin has been demonstrated to reverse the meiosis-deficient phenotype in aged mouse via SIRT1/SOD2, which is thought to be a major antioxidant enzyme in oocytes [27]. SIRT2 is a key effector of oocyte meiosis, and SIRT2-controlled histone H4K16 deacetylation is essential for oocyte meiosis. Li et al. revealed that melatonin could improve oocyte quality in aged mice through the SIRT2-dependent H4K16 deacetylation pathway [28]. Also, melatonin could counteract mitochondrial oxidative damage via SIRT3/FOXO3a pathway. As a ROS-regulated transcription factor, FOXO3a has been proved to significantly decrease the level of 8-hydroxydeoxyguanosine (8-OHDG) in mitochondria to protect mitochondrial function and reduce oxidative stress damage in mouse oocytes [29].

To the best of our knowledge, clinical trials on melatonin in human ovarian aging were not retrieved among the few common sites including http://ClinicalTrails.gov/. But a high-quality study on mice may indicate administration of melatonin could be a promising candidate for preventing ovarian aging [23]. In this research, mice were fed with water that contains melatonin (100 *μ*g/mL) from 13 weeks to 43 weeks and the number of ovulated oocytes, fertilization rate, and blastocyst rate were all significantly higher in the melatonin-supplemented mice than in controls. Tested by flow cytometry, another two-month-long trial also revealed that the percentage of ovarian cells in the G0-G1 phase was remarkably lower in the melatonin-treated rats (150 *μ*g/100 b.w., s.c. daily) compared to the control group, and conversely, the percentage of the S phase was higher [30]. With the exclusion of tumors, higher S phase implies that melatonin administration can increase the proliferative capacity of the ovarian cells at the decline of the reproductive function.

**Figure 1:**
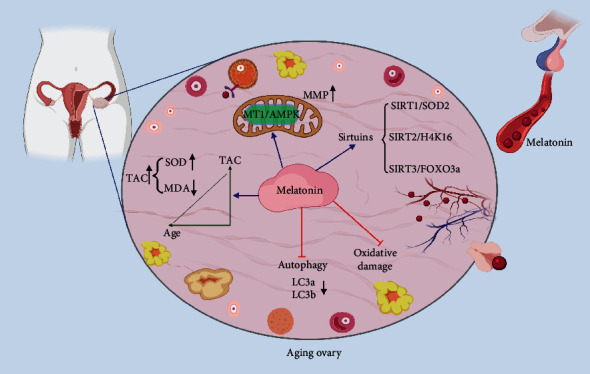
Melatonin delays ovarian aging. Melatonin, which is secreted by the pineal gland, exists in all stages of the oocyte and exerts its antioxidant function to delay ovarian aging. The activity of antioxidants often reflects the degree of ovarian aging. Melatonin increases total antioxidant capacity (TAC) and superoxide dismutase (SOD) levels in the ovary to counteract oxidative stress caused by ROS. At the same time, the level of malondialdehyde (MDA) is reduced. Melatonin also reduces oxidative stress damage by maintaining mitochondrial membrane potential through the MT1/AMPK pathway and controlling autophagy levels via reducing the expression of autophagy-related genes. Several pathways between melatonin and the anti-aging such as sirtuins have also been reported and confirmed in the ovarian aging aspects. TAC: total antioxidant capacity; MDA: malondialdehyde; SOD: superoxide dismutase; ROS: reactive oxygen species; MMP: mitochondrial membrane potential; LC3: light chain protein 3; MT1: melatonin receptor 1; AMPK: adenosine 5′-monophosphate- (AMP-) activated protein kinase; SIRT: sirtuin; FOXO3: forkhead box O3.

Altogether, melatonin plays an important role in combating oxidative stress, controlling autophagy, maintaining mitochondrial function, activating anti-aging genes, and improving fertility (Figure 1).

## 3. Melatonin Benefits Vitrification Freezing

Vitrification freezing is a technique that transforms cells into a vitrification state by adding a cryoprotectant and cooling them in an ultrafast manner. And the vitrification freezing of oocytes has become an important clinical method of female fertility preservation [31]. However, the extreme conditions of vitrification freezing and the toxicities of cryoprotectants, as well as the high lipid content of the oocytes themselves, will inevitably cause damage to the frozen oocytes. For example, during oocyte vitrification freezing, the freezing solution may disrupt the oocyte cell membranes and damage the mitochondria [32–34]. A prospective cohort study showed that an increase in the perivitelline gap as well as a thickening and hardening of the zona pellucida was observed in oocytes after vitrification freezing [35]. Also, reduced ATP content and inadequate mitochondrial energy can lead to impaired spindle assembly and decreased spindle density [36, 37]. The stability of the MMP and calcium level is particularly important for the quality of the oocyte. As a result of high levels of ROS, dysregulation of Ca^2+^ homeostasis can be triggered, thus resulting in a greater calcium load [38–40]. And other researchers have observed a significant decrease of MMP in human oocytes after recovery [41, 42]. Concurrently, altered MMP can induce the release of cytochrome C, which leads to a caspase cascade reaction and triggers apoptosis [43].

Acting as efficient free radical scavengers and antioxidants, melatonin and its metabolites can significantly improve the series of oxidative stress events in vitrification freezing and enhance the developmental capacity of frozen oocytes and embryos [33, 37]. These benefits further increase the oocyte fertilization rates and blastocyst formation rates in ART [44, 45]. A study in bovine oocytes pointed out that cryoprotectant containing 10^−9^ mol/L melatonin significantly decreased ROS levels, along with Ca^2+^ levels. It also showed that melatonin supplementation could inhibit mitochondrial-mediated apoptosis by decreasing *Bax* and *Bcl2* [44]. Meanwhile, in mice, melatonin could enhance the developmental potentials after vitrification freezing as it protects free radical damage, increases cytoplasmic and nuclear maturation, and upregulates maternal-to-zygotic transition (MZT) related gene expression [33]. Another research group illustrated that melatonin could promote mouse oocyte development by increasing the expression of GSH to maintain MMP and normal meiosis [37]. A summary of the ameliorative effects of melatonin on vitrification freezing damage is shown in Table 1.

**Figure 2:**
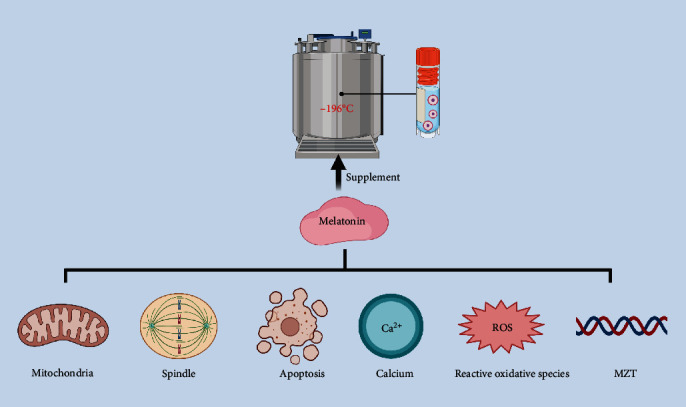
Melatonin benefits vitrification freezing. Cryoprotectant addition of melatonin significantly alleviates oxidative stress damage during vitrification freezing. Melatonin can mitigate oxidative stress damage either by direct antioxidation or by increasing the activity of antioxidant enzymes. Moreover, melatonin can maintain normal spindle morphology, decrease mitochondrial membrane potential, and keep intracellular Ca^2+^ homeostasis. Besides that, melatonin alters the apoptosis genes and the maternal-to-zygotic transition (MZT) genes.

The potential benefits of melatonin in vitrification freezing are invaluable for those with fertility preservation needs (Figure 2). As far as we are aware, no prospective studies of melatonin supplementation during the vitrification freezing of human oocytes have been reported. High-quality prospective randomized controlled trial (RCT) studies are needed to confirm the security and validity of melatonin in clinical cryopreservation.

## 4. Melatonin Promotes *In Vitro* Oocyte Maturation

IVM is a cutting-edge technique in the field of *in vitro* fertilization (IVF), in which immature eggs are removed from the ovaries after the application of ovulation stimulating drugs and then are *in vitro* cultured from GV or pre-GV stage to MII stage [46, 47]. Utilization of immature oocytes is an important component of ART [48]. However, excessive ROS caused by *in vitro* culture poses a marked challenge to oocyte quality and developmental potential. A series of oxidative stress-related injuries can lead to decreased oocyte developmental potential and increased cell apoptosis [38, 39]. The accumulation of higher than physiological amounts of oxygen radicals during IVM often causes the following damaging effects: (1) adverse outcomes such as DNA fragmentation, enzyme inactivation, and cell death [49]; (2) decreased MMP [9]; (3) increased Ca^2+^ levels [38, 40]; (4) impaired mitochondrial function, thus leading to a meiotic block and maturation disorders in oocytes [50, 51].

**Figure 3:**
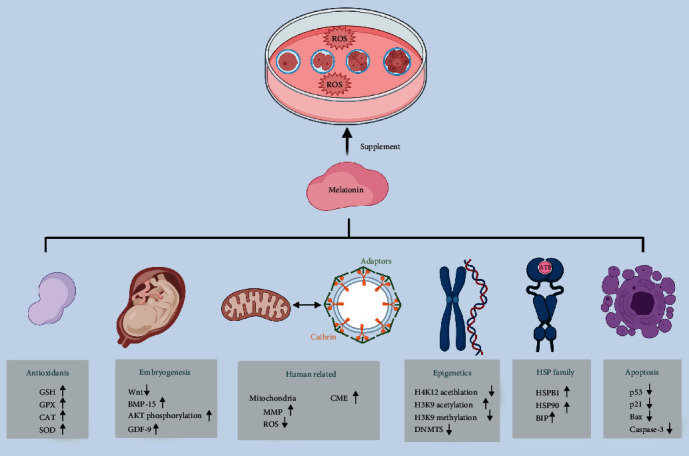
Melatonin improves IVM quality. Melatonin involves the process of epigenetic regulation, embryonic development, apoptotic, and antioxidant gene expression. The expression levels of the heat shock protein family are also significantly increased by melatonin supplementation. In human oocyte-related IVM experiments, melatonin improves IVM outcome by enhancing CME and by maintaining oocyte mitochondrial function. DNMTS: DNA methyltransferases; Wnt: canonical Wnt/*β*-catenin pathway; AKT: serine/threonine-specific protein kinase; BMP-15: bone morphogenetic protein 15; GDF-9: growth differentiation factor 9; CAT: catalase; SOD: superoxide dismutase; GSH: glutathione; GPX: glutathione peroxidase; HSPs: heat shock family proteins; CME: clathrin-mediated endocytosis.

The benefits of melatonin for IVM include several aspects, such as receptor activation, epigenetic modification, gene expression regulation, heat stress protection, and apoptosis suppression (Figure 3). Melatonin performs a variety of biological functions by activating MT1 and MT2 receptors in oocytes and granulosa cells to inhibit the activity of adenylate cyclase [52, 53]. In the regulation of epigenetic modifications, reduction of DNA methyltransferase expression rendered by melatonin leads to a decrease in overall DNA methylation level [54]. In addition, acetylation of histone H4K12 could be significantly decreased by melatonin during IVM [55]. For histone H3K9, melatonin can not only increase the overall H3K9 acetylation level but also decrease the H3K9 methylation level [56]. When considering embryonic development-related genes, melatonin can promote oocyte development by negatively regulating the classical Wnt pathway [57], increasing the phosphorylation of AKT, and upregulating bone morphogenetic protein 15 (BMP-15) and growth differentiation factor 9 (GDF-9, [58]). In addition, melatonin supplementation significantly increased the transcript levels of relevant antioxidants, including catalase (CAT), SOD, GSH, and GPX [59]. Reports on heat shock family proteins (HSPs) have confirmed that HSPB1 and HSP90 are significantly increased with melatonin supplementation, which subsequently promotes *in vitro* oocyte maturation [60]. The expression of heavy-chain binding protein (BIP), which also belongs to HSPs, was also increased after melatonin supplementation to further alleviate endoplasmic reticulum stress [61]. During the suppression of apoptosis, melatonin was involved in regulating *P53*, *Bax*, *P21*, and caspase-3 [62].

Melatonin exhibited many benefits during human oocyte IVM, but study concerning the mechanism of melatonin on human oocytes was limited. Our previous studies have suggested that melatonin enhances clathrin-mediated endocytosis (CME) through direct antioxidant effects. The increased expression of clathrin and adaptor protein-2 (AP2) can lead to the decreased level of cAMP, which is essential for maintaining the meiotic blockade and thus promoting human oocyte maturation [63]. Evidence also showed that melatonin can regulate mitochondria function to improve oocyte quality. In 2020, Zou et al. proved that the addition of melatonin at a concentration of 10^−5^ mol/L during the *in vitro* culture of human oocytes significantly increased the maturation rate of oocytes at the GV and MI stages as well as increasing the rate of high-quality blastocysts [9]. Even more gratifying, three healthy babies were born soon after that [9]. Although physiological concentrations of ROS are necessary for meiosis [64], low doses of melatonin treatment can significantly reduce the damage of oocytes due to additional ROS and oxidative stress. This implies the need for innovative improvements in the IVM routine, as well as IVM after vitrification cryoresuscitation.

## 5. Melatonin Improves the Dysregulation of Oxidative Stress Homeostasis Associated with PCOS

Representing pathological redox steady-state imbalance, PCOS is characterised of anovulatory infertility and hyperandrogenemia in women of reproductive age, accompanied with elevated oxidative stress in both serum and follicular fluid (FF). Numerous studies have found that decreased oocyte qualities in PCOS patients are associated with increased oxidative stress levels in FF [65]. We recently proposed that oxidative markers in FF, especially MDA and total oxidant capacity (TOC), were more closely correlated with embryo quality in *in vitro* fertilization and embryo transfer (IVF-ET) [66]. Indeed, women with PCOS tend to have higher lipid peroxides in the serum and FF. And these excess lipid peroxides produce excess MDA, which leads to DNA damage even apoptosis in oocytes [67]. Higher levels of oxidative stress may also lead to spindle disorders, organelle degeneration, cytoplasmic heterogeneity, and reduced mitochondrial function in oocytes [36, 39, 40, 68]. And ultimately, it results in poorer fertilization rates, lower high-quality blastocyst rates, and worse clinical pregnancy outcomes in PCOS patients [69]. Tamura's work reconfirms this conclusion that lower concentrations of melatonin in the FF can lead to oxidative stress homeostasis imbalance and follicular damage [70], resulting in decreased oocyte quality and ovulation difficulties [71]. Although serum melatonin levels are elevated in PCOS patients, this may simply be a compensatory response to inadequate ovarian melatonin levels [70, 72].

From the perspectives of antioxidant function, melatonin may influence some enzyme activity involved in metabolizing potentially reactive species to harmless molecules or inducing the synthesis of other endogenously produced antioxidants such as SOD, GPX, and glutathione reductase [73]. For instance, melatonin can improve oocyte maturation disorders caused by GSH deficiency in PCOS patients to some extent [36, 70]. And in terms of specific parameters of PCOS such as irregular menstrual cycles and hyperandrogenism, melatonin also possesses a protective effect. Firstly, melatonin is associated with abnormal ovulation in PCOS patients. In the ovary, melatonin binds to its corresponding receptor and promotes luteinization and ovulation as well as luteinizing hormone receptor expression and estrogen production [70, 74, 75]. Large amounts of ROS and reactive nitrogen species (RNS) are produced during ovulation, affecting the quality of oocytes [76]. Both melatonin and its metabolites can quench ROS and RNS thus inducing the expression of SOD and GPX [72, 76]. Meanwhile, melatonin levels are higher in preovulatory follicles and can significantly raise before ovulation [70]. These effects can be understood as a response to the excess oxidants that accumulate in the FF. Pacchiarotti et al. demonstrated that the oral administration of melatonin does increase the content of melatonin in the FF and has a synergistic effect with inositol to promote oocyte development and follicular discharge [75]. Besides, ovulation is often associated with local inflammatory responses [72], while melatonin can relieve inflammation by downregulating nuclear factor kappa-B (NF-*κ*B) [77]. With regard to inflammation, melatonin can also stimulate the release of the anti-inflammatory cytokines IL-4 and IL-10 and change the catalytic activity of myeloperoxidase (MPO) in neutrophils [77, 78]. Particularly, it can inhibit MPO's catalytic activity or directly remove hypochlorous acid (HOCL) [78]. Secondly, melatonin may improve hyperandrogenemia in PCOS patients. High concentrations of androgens can inhibit oocyte maturation, which can be rescued by melatonin [79]. The mechanism of androgen overproduction is caused by apoptosis of granulosa cell (GC) which can convert androgens into estradiol [79, 80]. Melatonin can upregulate B-cell lymphoma-2 (BCL-2) and downregulate B-cell lymphoma-2-associated X (BAX), thus preventing apoptosis of GC cells [79]. Besides, melatonin can reduce androgens by upregulating heme oxygenase 1 (HO-1), so as to rescue the inhibitory effects of androgens on oocyte maturation and development [79]. For the upstream raw material of androgen synthesis, melatonin significantly inhibits the expression of steroidogenic genes, leading to a reduction in androgen production [81]. Insulin resistance is also associated with excess androgens. Decreased brain and muscle ARNT-like protein 1 (BMAL1) contributes to insulin resistance via glucose transporter 4 (GLUT4), and a reduction in period (PER) 1 and PER2 promotes androgen excess via insulin-like growth factor-binding protein 4 (IGFBP4) and sex hormone-binding globulin (SHBG) in the liver. Melatonin treatment alleviated the hyperinsulinemia and hyperandrogenism of darkness-treated rats via BMAL1, PER1, and PER2 [82].

In view of the effects of melatonin, more and more clinical trials are being conducted on the therapeutic aspects of melatonin. Several high quality RCT studies have shown that orally melatonin administration at 3-5 mg daily, whether or not combined with other drugs such as folic acid and inositol, significantly increases the concentration of melatonin in serum and FF, thereby improving the quality of mature oocytes and increasing the clinical pregnancy rate in subsequent ART cycles [75, 83–86]. In the case of PCOS patients, multiple double-blinded RCT results also indicate that melatonin administration plays a positive effect and lead to better assisted reproductive outcomes by modulating the activity of some enzymes such as antioxidant enzymes and aromatase, regulating lipid metabolism, improving endocrine hormone levels like raising FSH, lowering LH and androgen levels, and relieving insulin resistance, as well as reducing inflammatory states [75, 85–90]. More specific information is detailed in Table 2.

Achieving these therapeutic benefits of melatonin requires a safety assessment of melatonin. Short-term use of melatonin even in extreme doses is generally considered safe [91, 92]. In experimental animal studies, exogenously administered melatonin has been given in doses up to 800 mg/kg without any acute toxic effects while a single dose of 1–10 mg is considered standard in humans [93]. However, taking high doses of melatonin that do not follow the natural circadian rhythm, such as taking it during daytime hours, may increase subjective sleepiness and lead to daytime lethargy, which is the most common side effect [94]. Other mild adverse effects might include dizziness, headache, and nausea [94, 95]. Conclusions on the long-term safety of melatonin are limited by the general lack of randomized, double-blind, placebo-controlled studies. However, the rare data available suggest that there are no serious adverse effects even with long-term use. A follow-up cohort study including 59 adolescents with chronic sleep-onset insomnia investigated the long-term effects of exogenous melatonin (mean duration of administration = 3.1 years; mean dose = 2.7 mg). And the distribution of adverse events did not differ in frequency between melatonin and placebo-treated groups [96].

**Figure 4:**
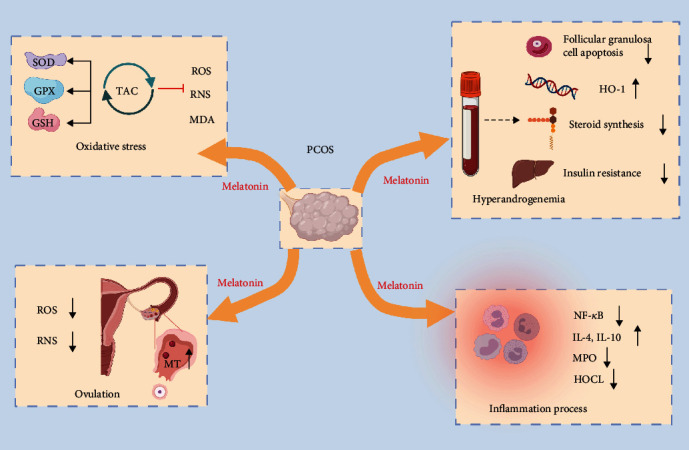
Melatonin regulates the homeostasis of the oxidative stress in PCOS. Melatonin benefits PCOS patients by reducing oxidative stress, promoting ovulation, modulating inflammation process, and reducing androgen production. It quenches ROS and RNS, increases the activity of antioxidant enzymes, and reduces intracellular malondialdehyde (MDA) levels. Melatonin also downregulates nuclear factor NF-*κ*B, stimulates the release of anti-inflammatory cytokines IL-4 and IL-10, and alters the catalytic activity of myeloperoxidase (MPO). In addition, the process of follicular discharge is associated with melatonin and elevated melatonin levels are observed in the preovulatory follicle. Finally, melatonin rescues the suppressive effects of granulosa cell induced hyperandrogenism on oocytes in a variety of ways, including upregulation of heme oxygenase 1 (HO-1), reduction of granulosa cell apoptosis, improvement of insulin resistance, and inhibition of steroidogenic gene expression. ROS: reactive oxygen species; RNS: reactive nitrogen species; MT: melatonin; SOD: superoxide dismutase; GSH: glutathione; GPX: peroxidase; MDA: malondialdehyde; NF-*κ*B: nuclear factor-*κ*-gene binding; IL: interleukin; HOCL: hypochlorous acid.

Taken together, melatonin can modulate redox homeostasis in PCOS by reducing oxidative stress, promoting ovulation, modulating inflammation process, and reducing androgen production (Figure 4). The administration of melatonin has been shown to significantly improve the PCOS prognosis [90]. However, the assessment of therapeutic use and safety in new areas beyond its traditional use still requires further validations via high-quality, multicenter prospective studies.

## 6. Summary

In summary, melatonin plays important roles in counteracting physiological ovarian aging, *in vitro* maturation, improving the quality of oocyte vitrification freezing, and reducing pathological oxidative stress imbalance in PCOS patients. Evidences showed that melatonin effectively and safely improves oocyte quality and reduces oxidative stress in reproduction, and it possesses well prospects for clinical applications. But whether long-term melatonin administration has side effects still needs to be demonstrated by high-quality RCT data. Meanwhile, the wide application of melatonin in clinical also requires high-quality, multicenter, and prospective clinical studies to provide more robust evidence especially in the field of *in vitro* fertilization.

## Figures and Tables

**Table 1:** Animal studies related to melatonin improvement in vitrification freezing.

Animals	Period	Concentration	Main results	Year	Ref
Mice	Oocytes (GV stage)	10^−7^ mol/L	↑ oocyte maturation, ↑MMP, ↑ATP, ↓ ROS, ↑GSH, ↑SAC-related genes	2019	[37]
Mice	Oocytes (MII stage)	10^−9^ mol/L	↑ ovarian cleavage rate and 4-cell embryo formation rate, ↑blastocyst development rate, ↑ MZT, ↓ ROS	2016	[33]
Mice	Oocytes (GV stage)	10^−11^ mol/L	↓ mitochondrial heat production, ↓ ROS level, ↑ mtDNA copy number, ↓ aneuploidy incidence	2019	[97]
Mice	Oocytes (MII stage)	10^−9^ mol/L	↑G1/S embryo percentage, ↓ ROS and GSH, ↓ P53, ↓ P21, ↓ E2F1	2018	[45]
Cattle	Embryos (Zygote)	10^−7^ mol/L	↑ ovarian cleavage rate, ↑ 8-cell embryo production rate, ↑ DNMT3A, ↑ OCC, ↑ CDH1, ↓ AQP3	2014	[98]
Cattle	Embryos (Two-cell)	10^−12^ mol/L	↑ ovarian cleavage rate, ↑ blastocyst formation rate, ↑ GSH, ↑ Bcl-X1, ↓ Bax	2014	[99]
		10^−9^ mol/L	↑ nourishment of the ectoderm, ↑ endocytosis number, ↑ GSH		
Cattle	Oocytes (MII stage)	10^−9^ mol/L	↓ ROS, ↓ mCa^2+^, ↓ Bax, ↓ Caspase-3 protein level, ↓ PS externalization rate, ↓ DNA fragmentation rate, ↑ MMP	2015	[44]
Rabbits	Embryos (Morula)	10^−3^ mol/L	↑ blastocyst development rate, ↑ GST activity, ↑ SOD activity, ↓ LPO, ↓ NO	2015	[100]

GV stage: maturation of the suppressed state of the germinal vesicle; MII: midsecond meiotic division; ROS: reactive oxygen species; GSH: glutathione; SAC genes: spindle assembly checkpoint-related genes; MZT: maternal to embryonic regulation-related genes; mtDNA: mitochondrial DNA; G1 phase: pre-DNA synthesis phase; S phase: DNA synthesis phase; P53, P21, E2F1: cell cycle-related genes; DNMT3A: DNA methyltransferase 3a; OCC: occluded protein; CDH1: calcium adhesion; AQP3: water channel protein 3; Bax: proapoptotic gene; Bcl-X1: B-cell lymphoma/leukemia-x long; mCa^2+^: mitochondrial Ca^2+^; PS: phosphatidylserine; GST: antioxidant enzyme glutathione-S-transferase; SOD: superoxide dismutase; LPO: lipid peroxidation; NO: nitric oxide.

**Table 2:** Results of clinical trials on melatonin administration in PCOS patients.

Country	Treatment	Duration	Main results	Sample	Year/Ref
Iran	Melatonin 3mg/day; from 3^rd^ day of menstruation to 10^th^ day; orally taken	7 days	Improved pregnancy rates	198	2019/[90]
Iraq	Melatonin 3mg/day 10 p.m.; orally taken	2 months	Reduced LH and BMI	50	2018/[101]
Italy	4 g/day myo-inositol, 3mg/day melatonin; orally taken	3 months	Increased the number of mature oocytes and the fertilization rate	46	2011/[85]
Iran	5mg melatonin supplements twice a day; orally taken	12 weeks	Reduced hirsutism, total testosterone, hs-CRP, and MDA; increased TAC and GSH levels; reduced gene expression of IL-1 and TNF-*α*	56	2019/[87]
Iran	5mg melatonin supplements twice a day; orally taken	12 weeks	Reduced insulin levels and HOMA-IR, and total- and LDL-cholesterol; increased QUICKI and gene expression of PPAR and LDLR	58	2019/[88]
Italy	Myo-inositol: 4000 mg, folic acid: 400 mcg, melatonin:3mg; orally taken from the first day of the cycle until 14 days after embryo transfer	14 days	Improved ovarian response to gonadotropin stimulation, with the result of better oocyte and embryo quality	165	2016/[75]
South Korea	800 μl IVM medium with 10 μmol /L melatonin	2 days	Improved immature oocytes maturation, as well as implantation and pregnancy rates	13	2013/[86]
Italy	Melatonin Fast 1 mg; 2 tablets a day; orally taken	6 months	Decreased androgens and AMH; increased FSH; improved menstrual disorders and hyperandrogenemia	40	2018/[89]

LH: luteinizing hormone; BMI: body mass index; IVF: *in vitro* fertilization; hs-CRP: high-sensitivity C-reactive protein; MDA: malondialdehyde; TAC: total antioxidant capacity; GSH: glutathione; IL-1: interleukin-1; TNF-*α*: tumor necrosis factor alpha; HOMA-IR: homeostasis model of assessment-insulin resistance; QUICKI: quantitative insulin sensitivity; LDL: low-density lipoprotein. Check index. PPAR-*γ*: peroxisome proliferator-activated receptor gamma; LDLR: low-density lipoprotein receptor; MI: myoinositol; FSH: follicle-stimulating hormone; AMH: anti-mullerian hormone.
